# Health-related quality of life is not impaired in children with undetected as well as diagnosed celiac disease: a large population based cross-sectional study

**DOI:** 10.1186/1471-2458-14-425

**Published:** 2014-05-05

**Authors:** Anna Myléus, Solveig Petersen, Annelie Carlsson, Solveig Hammarroth, Lotta Högberg, Anneli Ivarsson

**Affiliations:** 1 Department of Public Health and Clinical Medicine, Epidemiology and Global Health, Umeå University, Umeå, Sweden; 2 Department of Clinical Sciences, Child and Adolescent Psychiatry, Umeå University, Umeå, Sweden; 3 Department of Pediatrics, Clinical Sciences, Skånes University Hospital, Lund University, Lund, Sweden; 4 Pediatric Clinic, Norrtälje Hospital, Norrtälje, Swedens; 5 Department of Pediatrics in Norrköping, County Council of Östergötland, Norrköping, Sweden; 6 Department of Clinical and Experimental Medicine, Faculty of Health Sciences, Division of Pediatrics, Linköping University, Linköping, Sweden

**Keywords:** Celiac disease, Children, Health related quality of life, Kidscreen, Screening

## Abstract

**Background:**

Knowledge regarding the health-related quality of life (HRQoL) of children with celiac disease remains limited and inconclusive. We investigated the HRQoL of three groups of 12-year-olds with: *i)* undetected celiac disease *ii)* clinically diagnosed celiac disease, and *iii)* without celiac disease.

**Methods:**

A school-based cross-sectional multicenter screening study invited 18 325 children, whereof 68% consented to participate. Participants provided a blood sample, which was later analyzed for anti-tissue-tranglutaminase antibodies, and alongside filled in a questionnaire. When anti-tissue-tranglutaminase antibodies were elevated, a small intestinal biopsy verified the screening-detected celiac disease diagnosis. Self-reported HRQoL was measured using Kidscreen, a generic 52 items instrument with proven reliability and validity. Scores were linearly transformed into a 0–100 scale with higher values indicating better HRQoL. Mean values with standard deviations (mean ± SD) were compared, and uni- and multivariate logistic regression models tested the odds of a low HRQoL among children with undetected or diagnosed celiac disease, respectively.

**Results:**

Children with undetected celiac disease (n = 238) reported similar HRQoL as children without celiac disease (n = 12 037) (83.0 ± 11.0 vs. 82.5 ± 11.3, P = 0.51), and also similar HRQoL (82.2 ± 12.2, P = 0.28) to that of children with diagnosed celiac disease (n = 90), of whom 92% were adherent to treatment. Having undetected celiac disease did not increase the odds of low overall HRQoL, independent of sex, area of residence, study year and occurrence of gastrointestinal symptoms (adjusted odds ratio 0.77, 95% CI 0.54-1.10). Comparable results were seen for diagnosed celiac disease cases (adjusted odds ratio 1.11, 95% CI 0.67-1.85).

**Conclusion:**

Children with undetected celiac disease reported comparable HRQoL as their peers with diagnosed celiac disease, and those without celiac disease, when reporting prior to receiving the diagnosis through screening. Thus, children with celiac disease, both untreated and diagnosed, perceive their HRQoL as unimpaired by their disease.

## Background

Celiac disease is one of the most common chronic diseases in childhood, affecting around 1% of the population [1]. In some genetically predisposed individuals, dietary intake of gluten, found in wheat, ray, and barley, results in an immune-mediated small intestinal enteropathy [1-3]. Although the classical symptoms are related to the enteropathy (diarrhea, malnutrition, and failure to thrive), it has become evident that celiac disease can develop at any age with varying clinical presentations [4,5]. Especially when untreated, the disease has been associated with an increased risk of morbidity and mortality [3,6,7]. Serological markers indicative of untreated disease are in general use, but demonstration of small intestinal enteropathy remains the gold standard for diagnosis [8]. With treatment, a strict gluten-free diet, the serological markers normalize, the small intestinal mucosa recovers, and symptoms alleviate [3,9]. However, adhering to a strict gluten-free diet poses difficulties in everyday life including increased costs, social restrictions and stigmatisation, which may impact negatively on the health-related quality of life (HRQoL) [10-14].

Screening studies have shown that the majority of children with celiac disease are undiagnosed [4,15,16], and mass screening might be an option for finding these cases [17]. The high rate of under-diagnosis has been attributed to the varying clinical presentation, where the classical gastrointestinal symptoms most commonly are either mild or lacking [9,18]. Children with undiagnosed celiac disease do not experience enough symptoms to receive correct diagnosis; however, it remains poorly understood whether these children are completely unaffected or if undiagnosed celiac disease affects the HRQoL.

HRQoL assessments have become increasingly important in health care and research as it offers a more comprehensive understanding of the disease impact for the individual compared to that of laboratory analyses and disease related symptoms alone [19]. HRQoL is defined as a multidimensional construct encompassing several areas of life; physical, emotional, mental, social and behavioural components of functioning and wellbeing, as perceived by the individual and/or others [20,21]. Several HRQoL instruments have been developed during the last decades, including a European cross-country instrument called Kidscreen [21,22].

In previous studies among adults, undetected celiac disease has been associated with reduced HRQoL, mostly with a subsequent improvement after initiation of treatment [14,23]. Such an improvement has also been seen among screening-detected cases [24,25], although the findings are inconsistent [14,23,24,26,27]. Most previous studies of HRQoL and celiac disease have been performed among adults and studies of HRQoL in children with celiac disease are limited and inconclusive [23,28-31].

In this study we investigated and compared the HRQoL assessed by the generic Kidscreen instrument in three groups of 12-year-olds with: *i)* undetected celiac disease at the data collection, who thereafter received the diagnosis through screening, *ii)* clinically diagnosed celiac disease, and *iii)* children without celiac disease.

## Methods

### Design, procedure and study population

This study emanated from a school-based, cross-sectional, screening study entitled ETICS (Exploring the Iceberg of Celiacs in Sweden), which has previously been described in detail elsewhere [16,32,33]. In brief, the study invited all children in grade six (n = 18325; age ~12 years) attending schools in and around the five major Swedish cities Lund, Växjö, Norrköping, Norrtälje and Umeå during two one-year periods starting in September 2005 and 2009, respectively. At enrollment, the parents reported whether their child had celiac disease diagnosed through routine clinical care prior to the study. All children were invited to provide a blood sample and alongside to fill in a questionnaire. Data collection was performed in the schools by research nurses with assistance from school health nurses and teachers [16,29]. Afterwards, blood samples were analyzed for serological markers; anti-tissue-transglutaminase antibodies of IgA-type (tTG) for all children and for some also anti-endomysial antibodies IgA (EMA), or in case of IgA-deficiency serological markers of IgG type. Children with elevated markers (tTG >4 U/mL, or tTG 2–4 U/mL combined with EMA ≥1:5), but no previous diagnosis of celiac disease, were recommended a small intestinal biopsy at a close-by pediatric clinic [16,32]. Criteria for diagnosis were marked enteropathy (Marsh III), or the combination of milder enteropathy (Marsh I-II), HLA-DQ2 and/or DQ8 haplotype (genetic predisposition), symptoms and/or signs compatible with celiac disease, and clinical response to a gluten-free diet. Parental report of diagnosed celiac disease was confirmed through medical records and/or the National Swedish Childhood Celiac Disease Register following the same criteria.

The child’s celiac disease status at data collection was classified as ‘undetected celiac disease’ if the analyses showed a biopsy fulfilling the celiac disease criteria, i.e. the child received the diagnosis through the screening. To be classified as having ‘diagnosed celiac disease’ the parental report was confirmed with a diagnosis fulfilling also our criteria. Children with normal serological markers were classified as ‘without celiac disease’. In case of elevated serological markers in the screening but without biopsy-verified diagnosis (Marsh 0, Marsh I-II without other requirements, or small intestinal biopsy not performed) children were classified as having ‘potential celiac disease’.

**Figure 1:**
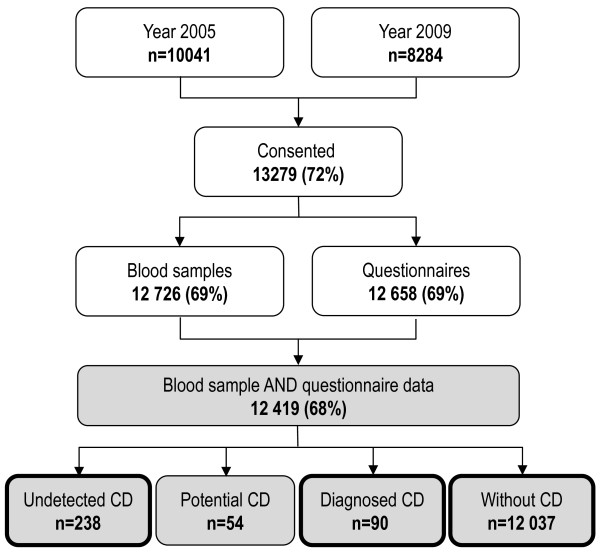
**Participants in the study.** The number of participating children with both a blood sample available for analyses of celiac disease (CD) serological markers, and a returned questionnaire (shaded grey). Included children are further divided into groups according to their CD status; undetected CD later receiving the diagnosis through the screening, potential CD i.e. elevated serological markers in the screening but without biopsy-verified diagnosis, diagnosed CD prior to the study treated with gluten-free diet, and children without CD. Three groups are included in the analyses (bold line).

A total of 13 279 (72%) parents and children gave informed consent to participate, and 12 419 (68%) children provided both blood samples and questionnaire data and were included in the current study (Figure 1). The study was approved by the Regional Ethical Review Board at Umeå University.

### Measures

#### Questionnaire

HRQoL was assessed by the generic self-report version of the 52 item Kidscreen instrument [20-22]. This instrument measures overall HRQoL along with 10 HRQoL subdomains capturing: physical well-being, psychological well-being, moods and emotions, self-perception, autonomy, parent relation and home life, financial resources, social support and peers, school environment, and social acceptance (bullying). Items utilize a 5-point response scale and a four week recall time. As recommended, scores were reversed and linearly transformed into a 0–100 scale with higher values indicating better HRQoL, and mean scores were estimated for all domains, given no more than one item were missing within the domain [20]. An exception was domains with only three items, i.e. financial resources and social acceptance, for which no missing were allowed. In the current study, items with multiple responses (n = 1053, 0.16%) were recoded as the mean if two adjacent responses, while other cases of multiple responses were handled as missing. Overall HRQoL was only estimated for children with available scores within all 10 subdomains. Domain scores were described as low if below the lowest quartile (≤25 percentile among the children without celiac disease) for the specific domain. The Kidscreen instrument has demonstrated acceptable levels of reliability and validity among children and adolescents in several countries including Sweden [21,22]. In this study population, the internal consistency was satisfactory (Cronbach’s alpha >0.7 for all domains) both among children with celiac disease and those without.

Celiac disease associated gastrointestinal symptoms were measured by additional questions capturing eight symptoms: nausea, stomach ache, upset stomach, abdominal gas, bloating, hard stools, loose stools and poor appetite [34]. All responses were given on a 5-point scale ranging from never to always over the past six months. Presence of gastrointestinal symptom(s) was defined as having one or more symptom often or always.

#### Blood samples for estimation of adherence

For children with diagnosed celiac disease blood samples were analyzed for serological markers as estimation of adherence with the recommended treatment (gluten-free diet). The serological marker tTG were determined by enzyme-linked immunosorbent assay in accordance with manufacturer’s instructions (Celikey, Phadia GmbH, Freiburg, Germany) [16]. In these children, tTG >5 U/mL was interpreted as non-adherence.

### Statistical analyses

Microsoft Access 2010 (Microsoft, Redmond, WA) was used for handling the ETICS database and PASW Statistics versions 20 (SPSS Inc, Chicago, IL) to perform statistical analyses. Descriptive information was given as proportions and mean values with standard deviations (±SD) or total range. Internal non-response for each subdomain were excluded, resulting in different number of children in each respective analyses (undetected celiac disease n = 227-237, diagnosed celiac disease n = 87-90, and children without celiac disease n = 11 517–11 963). Differences of HRQoL domain scores between children without celiac disease and undetected or diagnosed celiac disease, respectively, were tested by the Mann–Whitney U-test. This test was performed in the whole group and as well as after stratifications. Comparisons between proportions were performed by Chi-square test. Univariate and multivariate logistic regression models tested the odds of a low HRQoL score among children with undetected or diagnosed celiac disease compared to children without celiac disease. Multivariate models were adjusted for sex, study year (2005 or 2009), geographical area of residence (five areas), and the presence of gastrointestinal symptom(s) (yes or no). Statistical significance was defined as a two-tailed P < 0.05 corresponding to an odds ratio (OR) with a 95% confidence interval (CI) not including 1. For significant findings, the size of the difference was estimated using Cohen’s d, defined as the difference in mean between the groups divided by the pooled SD, and interpreted as small if below 0.20, moderate around 0.50 and large above 0.80 [35].

## Results

### Characteristics of the participants

Of the 12 419 participating children with both blood samples and questionnaire data, 292 children without diagnosed celiac disease presented elevated serological markers. Of these 13 declined further investigation with small intestinal biopsy and 41 had a normal biopsy, leaving 238 children (137 girls) with undetected celiac disease at data collection (Figure 1). The 54 children with potential celiac disease were not further analysed in this sub-study. Among the remaining, 90 children (61 girls) were classified as having diagnosed celiac disease and they had been all recommended a gluten-free diet. Mean age (±SD) at diagnosis was 4.7 ± 4.0 years and the majority (n = 83, 92%) had normal tTG at data collection. Seven children revealed elevated tTG, indicating non-adherence with the gluten-free diet (range 9.0-30 U/ml). They were not handled separately, since they were few and there was no correlation between tTG and HRQoL (r^2^ = 0.24).

In Table 1 the characteristics of the participants stratified by celiac disease status is summarized. In 2005, a larger number of children were invited than in 2009 due to declining birth rates in Sweden. The largest study site was Lund [32]. Children with undetected celiac disease reported gastrointestinal symptoms equally commonly as the children without celiac disease (21.1% vs. 21.8%, P = 0.80). Of the children with diagnosed celiac disease 22.2% reported symptom (Table 1).

**Table 1:** Characteristics of the participating children stratified by celiac disease (CD) status

**Characteristics (%)**	**Undetected CD**^**1**^	**Diagnosed CD**^**2**^	**Without CD**
	**N = 238**	**N = 90**	**N = 12 037**
Sex			
*Girls*	58	68	49
*Boys*	42	32	51
Year			
*2005*	63	66	57
*2009*	37	34	43
Geographical area of residence			
*Lund*	42	48	44
*Växjö*	20	14	14
*Norrköping*	11	17	12
*Norrtälje*	13	4	11
*Umeå*	14	17	18
Gastrointestinal symptoms			
*No*	79	78	78
*Yes*	21	22	22

^1^At the data collection CD was undetected, later diagnosed through screening.

^2^CD diagnosed through routine clinical care prior to the study.

### HRQoL in children with celiac disease, undetected or diagnosed

In general, the children reported relatively high HRQoL (mean ± SD: 82.5 ± 11.3), with functioning and wellbeing in the 10 HRQoL subdomains ranging from a mean score of 76.4 ± 17.1 (school environment) to 92.0 ± 14.7 (social acceptance).

**Figure 2:**
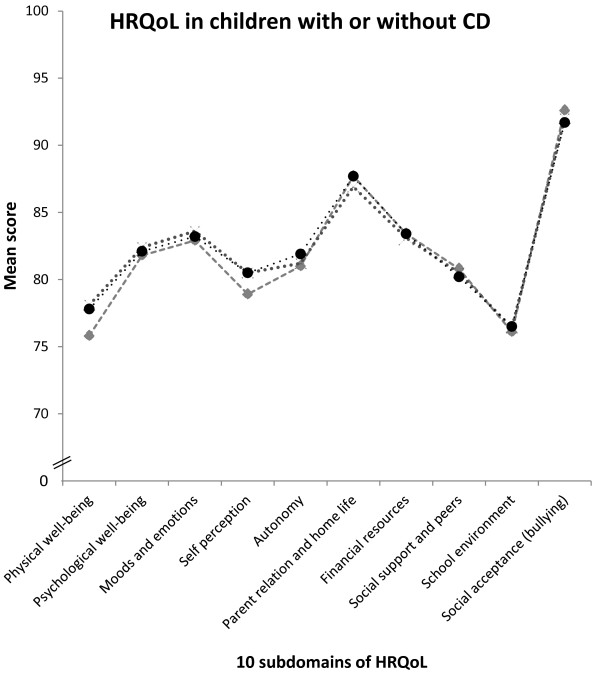
**HRQoL in children with and without celiac disease.** Health-related quality of life (HRQoL) profiles assessed with the Kidscreen-52 subdomains for the three groups of children; with undetected or diagnosed celiac disease (CD), and without CD (non-CD). We observed no statistically significant difference in any subdomain.

Children with undetected celiac disease reported comparable overall HRQoL as their peers without celiac disease (mean ± SD: 83.0 ± 11.0 vs. 82.5 ± 11.3, P = 0.51), and there was no difference between children with undetected and diagnosed celiac disease either (mean ± SD: 83.0 ± 11.0 vs. 82.2 ± 12.2, P = 0.28). These findings were replicated in all HRQoL subdomains (Figure 2). Details are found in Additional file 1: Table S1. Moreover, having undetected celiac disease did not increase the odds of low overall functioning and wellbeing, independent of sex, area of residence, study year and occurrence of gastrointestinal symptoms (adjusted OR 0.77, 95% CI 0.54-1.10). Similarly, we found no increased unadjusted or adjusted odds in any separate HRQoL subdomain (Table 2).

**Table 2:** Odds of low health-related quality of life (HRQoL) in children with undetected and diagnosed celiac disease (CD) compared with children without CD

**HRQoL**^**1**^	**Low HRQoL**	**Crude**	**Adjusted**
	**n (%)**^**2**^	**OR (95% CI)**^**3**^	**OR (95% CI)**^**4**^
Physical well-being			
*Without CD*^*5*^	3491 (29.6)	1	1
*Undetected CD*^*6*^	66 (28.3)	0.94 (0.71-1.26)	0.93 (0.69-1.27)
*Diagnosed CD*^*7*^	30 (33.7)	1.21 (0.78-1.88)	1.29 (0.81-2.06)
Psychological well-being			
*Without CD*	3501 (29.7)	1	1
*Undetected CD*	69 (29.6)	0.99 (0.75-1.32)	0.98 (0.73-1.34)
*Diagnosed CD*	28 (31.5)	1.09 (0.69-1.70)	1.14 (0.71-1.82)
Moods and emotions			
*Without CD*	3169 (26.8)	1	1
*Undetected CD*	58 (25.0)	0.91 (0.67-1.23)	0.85 (0.62-1.18)
*Diagnosed CD*	24 (26.7)	0.99 (0.62-1.59)	1.0 (0.61-1.65)
Self-perception			
*Without CD*	3372 (28.2)	1	1
*Undetected CD*	60 (25.5)	0.87 (0.65-1.17)	0.79 (0.58-1.09)
*Diagnosed CD*	32 (35.6)	1.41 (0.91-2.17)	1.15 (0.72-1.86)
Autonomy			
*Without CD*	3015 (25.2)	1	1
*Undetected CD*	49 (20.7)	0.77 (0.56-1.06)	0.74 (0.53-1.03)
*Diagnosed CD*	22 (24.7)	0.97 (0.60-1.58)	0.96 (0.57-1.60)
Parent relation and home life			
*Without CD*	2469 (20.8)	1	1
*Undetected CD*	45 (19.2)	0.91 (0.65-1.26)	0.85 (0.60-1.21)
*Diagnosed CD*	16 (18.0)	0.83 (0.49-1.44)	0.92 (0.52-1.60)
Financial resources			
*Without CD*	4048 (34.8)	1	1
*Undetected CD*	75 (33.0)	0.92 (0.70-1.22)	0.84 (0.63-1.13)
*Diagnosed CD*	31 (35.2)	1.02 (0.66-1.58)	0.94 (0.59-1.51)
Social support and peers			
*Without CD*	2403 (20.2)	1	1
*Undetected CD*	41 (17.4)	0.83 (0.59-1.17)	0.92 (0.65-1.30)
*Diagnosed CD*	19 (21.1)	1.06 (0.64-1.76)	1.30 (0.77-2.19)
School environment			
*Without CD*	2527 (21.9)	1	1
*Undetected CD*	44 (19.0)	0.84 (0.61-1.17)	0.84 (0.59-1.20)
*Diagnosed CD*	24 (27.6)	1.36 (0.85-2.17)	1.30 (0.77-2.18)
Social acceptance (bullying)			
*Without CD*	2499 (21.7)	1	1
*Undetected CD*	52 (22.6)	1.05 (0.77-1.44)	1.06 (0.76-1.47)
*Diagnosed CD*	14 (16.1)	0.69 (0.39-1.23)	0.72 (0.39-1.31)

^1^HRQoL was assessed using the Kidscreen-52 instrument.

^2^Low HRQoL was defined as the children reporting a score in the lowest quartile. Number and proportion of children reporting low HRQoL, excluding internal non-response for each respective subdomain.

^3^Univariate logistic regression giving the odds ratio (OR) with 95% confidence interval (CI).

^4^Multivariate logistic regression model including sex, phase of the study, geographical area, and presence of gastrointestinal symptoms.

^5^Children with normal serological markers (N = 12 037).

^6^At the collection of the HRQoL data CD was undetected (N = 238), later diagnosed through screening.

^7^CD diagnosed through routine clinical care prior to the study (N = 90).

Children with diagnosed celiac disease also reported similar overall HRQoL as children without celiac disease (mean ± SD: 82.2 ± 12.2 vs. 82.5 ± 11.3, P = 0.29). Again, the findings were replicated in all 10 HRQoL subdomains studied (Figure 2). Diagnosed celiac disease was not associated with low overall functioning and wellbeing. Adjusting for sex, area of residence, study year and gastrointestinal symptoms did not affect the finding (adjusted OR 1.11, 95% CI 0.67-1.85) and similar results were seen for all 10 HRQoL subdomains (Table 2).

**Figure 3:**
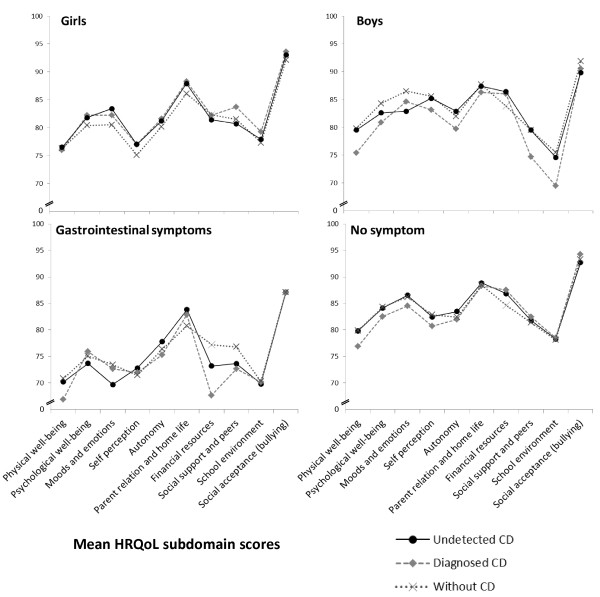
**HRQoL in children with and without celiac disease, stratified by sex and gastrointestinal symptoms.** Health-related quality of life (HRQoL) profiles by Kidscreen-52 subdomains for the three groups of children; with undetected or diagnosed celiac disease (CD), and without CD (non-CD) stratified by sex and presence of gastrointestinal symptoms (yes or no). Overall, girls and children with gastrointestinal symptoms reported lower HRQoL but we observed no difference between the three groups in any subdomain in any strata.

Considering the HRQoL stratified by sex showed no differences between children with and without celiac disease (Figure 3), although girls reported lower HRQoL than boys, except for the domains of social support and peers, and school environment which had the opposed relationship. Children reporting gastrointestinal symptoms also reported lower HRQoL than those without symptoms. Stratified analyses showed no HRQoL difference between children with and without celiac disease independent of the occurrence gastrointestinal symptoms (Figure 3). The study was performed in two phases and the HRQoL among the children was somewhat higher in 2005 compared to 2009, however, the effect size was neglectable (Cohen’s d <0.1) (Additional file 2: Table S2) and the two groups were handled as one.

## Discussion

In this large population based study in Sweden, children with undetected celiac disease reported similar HRQoL as children with diagnosed celiac disease. These children also reported HRQoL comparable to Swedish children without celiac disease. Accordingly, children with undetected or diagnosed celiac disease were not at increased odds for having low HRQoL. To the best of our knowledge, this is the first large population based study capturing the HRQoL of children with both undetected and diagnosed celiac disease by use of a comprehensive standardized HRQoL measure.

A previous population based study from our group, found no HRQoL difference between 153 school aged children with undetected celiac disease and children without celiac disease, when investigating self-reported HRQoL by the shorter six item EQ-5D-Y HRQoL instrument [29]. In the current study we confirmed these results in an extended study group (238 12-year old children with undetected celiac disease) and by use of a more comprehensive assessment of HRQoL (full-form 52 item Kidscreen instrument). These findings thus suggest that HRQoL is not impaired in school aged children with undetected celiac disease.

One study in younger children is however partly contradictive. This study found lower parent-reported HRQoL in children diagnosed through screening at age 2–4 years, but only among the children with gastrointestinal symptoms; children without symptoms had similar HRQoL as a population based reference group [36]. In our study, stratifying for gastrointestinal symptoms did not affect the results. The issue of gastrointestinal symptoms in children with celiac disease is however complicated. In a qualitative study from our group it was shown that school aged children diagnosed with celiac disease through screening in retrospect realized that they had experienced a varying degree of symptoms associated with the undetected disease, which they were not aware of prior to treatment [13]. Similarly, among children who were diagnosed with celiac disease through screening in at-risk groups, 50% of those with no symptoms at diagnosis reported alleviation of symptoms after one year of treatment [28], further indicating that unawareness of celiac disease related symptoms prior to treatment is common among children. This adaption to symptoms related to celiac disease in childhood may partly explain the lack of HRQoL impairment in children with undetected celiac disease when they report prior to knowledge of the disease.

Our findings of an unimpaired self-reported HRQoL in children with undetected celiac disease contributes to an understanding of why most celiac disease cases are not identified in clinical practice. Despite moderate to severe enteropathy [16,32], they are not presenting an affected overall HRQoL, and children with a low HRQoL do not have higher odds of undetected celiac disease than other children. Thus, mass screening using serological markers might be necessary to identify all cases of celiac disease. However, the issue of mass screening is still controversial and more research is needed before it can be determined whether it is an appropriate public health intervention or not [3,17].

In our study also children with diagnosed celiac disease reported HRQoL corresponding to that of children without celiac disease. This finding has been seen also in other studies [29,36,37], although previous findings are not conclusive. Inconclusive findings could originate from different methods for assessing HRQoL, especially when taking different aspects of HRQoL into account. Domains more linked to feeding and socialization have been shown to be more impaired due to the gluten-free diet [30,31,38]. In line with these findings we saw the highest odds for low HRQoL among children with diagnosed celiac disease in the subdomain school environment, although the difference was not statistically significant (Table 2). A celiac disease specific HRQoL instrument focuses in detail on aspects affected by the gluten-free diet and could therefore be more sensitive than a generic instrument [23,38]. Thus, we cannot exclude that our findings could, at least partly, be explained by a failure of the generic instrument Kidscreen to fully capture if diagnosed celiac disease affects HRQoL.

It has been shown that adherence with the gluten-free diet is important for obtaining similar HRQoL among children with celiac disease as their peers [39]. However, the burden of following a strict gluten-free diet has been associated with reduced HRQoL [40]. In our study, adherence with the gluten-free diet was high (92%) in comparison with other studies [14,31,41]. However, occasional gluten intake might not be captured by elevated tTG resulting in overestimation of the adherence. We were also limited by the cross-sectional study design where inference whether non-adherence affected HRQoL, or if children with low HRQoL due to other reasons did not manage to adhere to the gluten-free diet, could not be made. Therefore, we did not further investigate the influence of adherence on HRQoL in our study. Larger longitudinal studies investigating the influence of treatment adherence on HRQoL, and visa verse, are warranted.

A strength of our study was that it was based on an actual population-based screening study. Thus, the children with undetected celiac disease emanated from the general population, and in contrast to several other studies, we were able to assess the HRQoL prior to the diagnosis of screening-detected celiac disease [23,28]. Moreover, the children with diagnosed celiac disease were also identified from the general population, compared to the more common recruitment through patient societies [23]. As there may be a selection in who joins a patient society, the current strategy is likely to give a more accurate description of HRQoL in children with diagnosed celiac disease. The population-based strategy, however, also constituted a limitation. Albeit a relatively large cohort (n = 12 419 participants) the number of children with celiac disease were limited and we cannot exclude that lack of power affected our findings, especially after stratifications. The participants comprised 69% of all invited children in sixth grade. Their HRQoL were comparable to the that found by the Swedish National Institute of Public Health when they performed a countrywide, all-encompassing survey using the same HRQoL instrument, during the same time period, and in the same age group [42]. This suggests that our HRQoL results are representative for 12-year-old children in Sweden, and due to the homogeneity of the Swedish society, the association between HRQoL and celiac disease likely is the same across the country. The lack of adjustment for socioeconomic status may, however, constitute a potential limitation. Socioeconomic status is negatively associated with HRQoL [43], and might be associated with celiac disease, thereby constituting a potential confounder. However, the relationship between socioeconomic status and celiac disease has contradictory findings also from Sweden [44-46], with the largest study rejecting an association [47]. To the best of our knowledge, the only previous study which has tested the potential influence of socioeconomic status on HRQoL in celiac disease did not find an association between the two [48].

## Conclusion

Children with undetected and thereby untreated celiac disease reported comparable HRQoL as their peers when reporting prior to receiving the celiac disease diagnosis through screening. They also reported similar HRQoL as children with diagnosed celiac disease. Thus, children with diagnosed and treated celiac disease may be able to attain the same HRQoL as their peers without the disease. HRQoL encompasses several areas of functioning and wellbeing, and the current study suggests that children with celiac disease, both undetected and diagnosed, perceive unimpaired functioning and wellbeing within all such areas of HRQoL.

## Abbreviations

CI: Confidence interval; EMA: Anti-endomysial antibodies IgA; ETICS: Exploring the Iceberg of Celiacs in Sweden – screening study; HRQoL: Health-related quality of life; OR: Odds ratio; SD: Standard deviation; tTG: Anti-tissue-transglutaminase antibodies IgA.

## Competing interests

The authors declare that they have no competing interests.

## Authors’ contribution

All authors contributed to the conception, design and planning of the study, and to the data collection. AM performed the statistical analyses, interpretation of data, and wrote the first draft and the final manuscript. SP contributed to the statistical analyses, data interpretation, and writing of the manuscript. AI was principal investigator of the study. All authors reviewed the manuscript critically and approved its final version.

## Pre-publication history

The pre-publication history for this paper can be accessed here:

http://www.biomedcentral.com/1471-2458/14/425/prepub

## Supplementary Material

Additional file 1: Table S1 Details of findings presented in Figure 2. Click here for file

Additional file 2: Table S2 Comparisons of health-related quality of life (HRQoL) between data collections 2005 and 2009. Click here for file
