# Supplementary material for: Health-related quality of life is not impaired in children with undetected as well as diagnosed celiac disease: a large population based cross-sectional study
Source: BMC Public Health. 2014 May 5;14:425. doi: 10.1186/1471-2458-14-425 (PMC4021079; doi:10.1186/1471-2458-14-425)
Supplement: Additional file 1: Table S1 — Details of findings presented in Figure 2. [file 1471-2458-14-425-S1.pdf]

Additional table 1. Details of findings presented in Figure 2

Health-related quality of life (HRQoL)[1]	Mean±SD			Comparison[2]	
	Undetected CD[3] N=238	Diagnosed CD[4] N=90	Without CD N=12 037	P-value Undetected vs. without	P-value Diagnosed vs. without
Physical well-being	77.8±17.0	75.8±16.7	78.1±15.8	0.751	0.219
Psychological well-being	82.1±14.3	81.8±14.1	82.4±14.6	0.517	0.590
Moods and emotions	83.2±17.2	82.9±16.9	83.6±16.3	0.743	0.813
Self-perception	80.5±17.1	78.9±18.4	80.5±18.5	0.463	0.365
Autonomy	81.9±14.9	81.0±16.0	81.2±15.8	0.573	0.926
Parent relation and home life	87.7±13.0	87.7±14.4	86.9±14.8	0.935	0.769
Financial resources	83.4±19.8	83.4±20.9	83.1±20.3	0.968	0.518
Social support and peers	80.2±16.1	80.8±15.5	80.5±15.4	0.879	0.709
School environment	76.5±17.3	76.1±16.8	76.4±17.1	0.791	0.783
Social acceptance (bullying)	91.7±14.6	92.6±15.1	92.0±14.7	0.519	0.419

[1] HRQoL was assessed with the Kidscreen-52 instrument, comprising 10 subdomains of HRQoL.
[2] Comparison of groups using Mann-Whitny U-test, comparing the two CD groups with children without CD, respectively.
[3] At the collection of the HRQoL data celiac disease (CD) was undetected, later diagnosed through screening.
[4] CD diagnosed through routine clinical care prior to the study.
