# Supplementary material for: Health-related quality of life is not impaired in children with undetected as well as diagnosed celiac disease: a large population based cross-sectional study
Source: BMC Public Health. 2014 May 5;14:425. doi: 10.1186/1471-2458-14-425 (PMC4021079; doi:10.1186/1471-2458-14-425)
Supplement: Additional file 2: Table S2 — Comparisons of health-related quality of life (HRQoL) between data collections 2005 and 2009. [file 1471-2458-14-425-S2.pdf]

Additional table 2. Comparisons of health-related quality of life (HRQoL) between data collections 2005 and 2009.

**HRQoL**[1]	HRQoL in the ETICS-study mean±SD		Comparison of the years	
	2005[2]	2009[2]	P-value[3]	Cohen's d[4]
Physical well-being	77.7±16.0	78.5±15.5	0.005	0.05
Psychological well-being	81.9±14.8	83.1±14.5	<0.0001	0.08
Moods and emotions	83.3±16.4	84.0±16.3	0.001	0.05
Self-perception	80.5±18.0	80.4±19.0	0.212	-
Autonomy	80.8±15.6	81.6±16.0	<0.0001	0.05
Parent relation and home life	86.6±14.8	87.4±14.7	<0.0001	0.06
Financial resources	82.5±20.4	83.8±20.2	<0.0001	0.07
Social support and peers	80.1±15.3	81.0±15.7	<0.0001	0.06
School environment	75.5±16.9	77.6±17.3	<0.0001	0.12
Social acceptance (bullying)	91.9±14.9	92.2±14.4	0.570	-

[1] HRQoL was assessed with the Kidscreen-52 instrument, comprising 10 subdomains of HRQoL.
[2] Years of the data collection in the ETICS-study; 2005 n=7 152 and 2009 n=5 267
[3] Comparisons between the years using Mann-Whitney U-test.
[4] Cohen's d estimated as the difference in mean divided by the pooled SD, and interpreted as small if below 0.2.
